# Estimation of utility weights for human papilloma virus-related health states according to disease severity

**DOI:** 10.1186/s12955-016-0566-8

**Published:** 2016-11-28

**Authors:** Minsu Ock, Jeong-Yeol Park, Woo-Seung Son, Hyeon-Jeong Lee, Seon-Ha Kim, Min-Woo Jo

**Affiliations:** 1 Department of Preventive Medicine, University of Ulsan College of Medicine, 88, Olympic-Ro 43-Gil, Songpa-Gu Seoul, 138-736 South Korea; 2 Department of Obstetrics and Gynecology, University of Ulsan College of Medicine, Asan Medical Center, Seoul, South Korea; 3 Department of Nursing, College of Nursing, Dankook University, Cheonan, South Korea

**Keywords:** Cervical cancer, Human papilloma virus, Visual analogue scale, Standard gamble

## Abstract

**Background:**

A cost-utility study of a human papilloma virus (HPV) vaccine requires that the utility weights for HPV-related health states (i.e., cervical intraepithelial neoplasia (CIN), cervical cancer, and condyloma) be evaluated. The aim of the present study was to determine the utility weights for HPV-related health states.

**Methods:**

Hypothetical standardised health states related to HPV were developed based on patient education material and previous publications. To fully reflect disease progression from diagnosis to prognosis, each health state comprised four parts (diagnosis, symptoms, treatment, and progression and prognosis). Nine-hundred members from the Korean general population evaluated the HPV-related health states using a visual analogue scale (VAS) and a standard gamble (SG) approach, which were administered face-to-face via computer-assisted interview. The mean utility values were calculated for each HPV-related health state.

**Results:**

According to the VAS, the highest utility (0.73) was HPV-positive status, followed by condyloma (0.66), and CIN grade I (0.61). The lowest utility (0.18) was cervical cancer requiring chemotherapy without surgery, followed by cervical cancer requiring chemoradiation therapy (0.42). SG revealed that the highest utility (0.83) was HPV-positive status, followed by condyloma (0.78), and CIN grade I (0.77). The lowest utility (0.43) was cervical cancer requiring chemotherapy without surgery, followed by cervical cancer requiring chemoradiation therapy (0.60).

**Conclusions:**

This study was based on a large sample derived from the general Korean population; therefore, the calculated utility weights might be useful for evaluating the economic benefit of cancer screening and HPV vaccination programs.

**Electronic supplementary material:**

The online version of this article (doi:10.1186/s12955-016-0566-8) contains supplementary material, which is available to authorized users.

## Background

Cervical cancer is the fourth most common cancer among women world-wide; indeed, there were an estimated 266,000 deaths due to cervical cancer in 2012, accounting for 7.5% of all female cancer deaths [1]. Persistent infection with human papillomavirus (HPV), especially HPV types 16 and 18, is a risk factor of cervical cancer. Furthermore, HPV causes cervical intraepithelial neoplasia (CIN), anal, penile, vaginal, vulvar, and head/neck cancers, anogenital warts, and recurrent respiratory papillomatoses, leading to deaths in both genders [2].

The HPV vaccine was developed to prevent HPV. Many clinical trials show the efficacy of the HPV vaccine in this regard; it also prevents precancerous lesions and genital warts [3–8]. From an economic perspective, cost-effectiveness analyses continuously show that HPV vaccination of preadolescent girls and boys is good value for money [9]. Thus, many countries are implementing HPV vaccine programs at the national level; it is suggested that the HPV vaccination should be encouraged to reduce the overall burden of HPV-related disease [10].

When evaluating the efficacy of treatments for HPV-related diseases, both the clinical outcome and the health-related quality of life (HRQOL) should be taken into account. Several indicators of disease burden have been used, including quality adjusted life years (QALYs), which combines the number of years lived by an individual with their HRQOL. QALYs are used primarily to correct a patient’s life expectancy based on the HRQOL they are predicted to experience throughout their life-time (or part of it) [11]. QALYs are calculated by multiplying the length of time spent in a particular health state by the utility weight associated with that health state [12]. Thus, utility weights (or health utilities) are needed to calculate QALYs.

In particular, the HRQOL associated with HPV-related diseases such as cervical cancer needs to be assessed in light of increasing survival rates. For this reason, both cost-effectiveness and cost-utility studies are needed. To carry out cost-utility studies of HPV-related diseases, their utility weights must be calculated according to disease severity. Although some studies determined the utility weight for cervical cancer according to health state reflecting patient preferences [13–16], few studies have examined how members of the general population evaluate their own preferences in terms of cervical cancer or precancerous lesions [17, 18]. The utility weight for HPV-related diseases reflecting preferences of the general population is much more in need in various countries, because the perspectives of the general population provide more relevant information for comparative assessments of health interventions [19]. In addition, guidelines for economic evaluation of pharmaceuticals in Korea placed more emphasis on societal perspective than provider’s perspective or patients’ perspective [20]. Therefore, the aim of the present study was to determine the utility weights for HPV-related health states based on data derived from a sample of the general Korean population. These results will be used to perform a cost-utility study and to establish a national immunisation program for HPV-related diseases.

## Methods

### Participant selection

The target population comprised adults (≥19 years) living in Korea. A total of 900 representative individuals were randomly selected from this target population using a multistage stratified quota method. A sample quota was assigned to each of the 15 regions of Korea according to population and socio-demographic factors, including gender, age, and education level (as defined by the resident registration data (June 2013), available from the Ministry of Administration and Security, South Korea). The participants were recruited in the street along with quotas.

### HPV-related health states

A draft of the standardised HPV-related health states was created by two investigators (M Ock and MW Jo) based on patient education materials provided by Asan Medical Center (AMC), and on data from previous studies [13, 17]. One gynaecologic oncologist from AMC (Park JY) reviewed and modified the draft. Each HPV-related health state was designed to reflect a hypothetical scenario: HPV-positive status; CIN grade I; CIN grade II/III; cervical cancer requiring simple or radical hysterectomy; cervical cancer requiring radical hysterectomy and subsequent radiotherapy (and chemotherapy); cervical cancer requiring chemoradiation therapy; cervical cancer requiring chemotherapy without surgery; and condyloma (genital warts [condyloma acuminatum]) (Table 1). Each hypothetical scenario comprised four parts: diagnosis, symptoms, treatment, and disease progression and prognosis. The scenarios included diagnostic procedures, common physical side effects of treatment, emotional aspects of treatment, and information related to the possibility of complete recovery and 5-year survival rates. All eight hypothetical scenarios for the HPV-related health states are described in detail in Additional file 1.

**Table 1:** Eight hypothetical human papilloma virus-related health states

Number	Scenario
1	Human papilloma virus positive state
2	Cervical intraepithelial neoplasia grade I
3	Cervical intraepithelial neoplasia grade II/III
4	Cervical cancer requiring simple or radical hysterectomy
5	Cervical cancer requiring radical hysterectomy and subsequent radiotherapy (and chemotherapy)
6	Cervical cancer requiring chemoradiation therapy
7	Cervical cancer requiring chemotherapy without surgery
8	Condyloma (genital warts [condyloma acuminatum])

### Interviews

This study was approved by the institutional review board of AMC (S2014-1396-000) and all respondents consented to participating in the study. The survey was conducted using face-to-face computer-assisted interviews at respondents’ home. The interviewers were fully trained with respect to HPV-related health states and undertook both evaluation methods: a visual analogue scale (VAS) and a standard gamble (SG) approach. All interviewers practiced in pairs before conducting the field survey. In total, the interviewers received about 2.5 h of training.

Participants were initially asked about their gender, age, and educational level. Next, we elicited participants’ preference using two valuation methods: VAS and SG. We asked participants to imagine themselves in the HPV-related health states. In the case of female-specific health states, such as cervical cancer and CIN, we made male participants imagine a woman they were close to, such as mother or wife, suffering from these health states. Participants evaluated each of five health states, one of which was being dead, on a VAS scale. The other four health states were randomly selected from the eight hypothetical health states. Being dead state was the last scenario to be rated on the VAS scale. Each participant consecutively evaluated health states using the SG approach. Again five health states were randomly selected from the eight hypothetical states. The health states for the SG might or might not overlap with those of the VAS. After the two rounds of valuation, the participants were asked about other socio-demographic factors, such as employment and income. They were also asked whether they had undertaken an ambulatory care visit within the past 2 weeks, been hospitalised during the last 12 months, or had any morbidities. We defined morbidity as the presence of any disease in the participants and a list of diseases was not specifically provided. Visual aids were used to help respondents understand the questions. The procedure of computer-assisted personal interviewing is available in the Additional file 2.

### Valuation methods

We used VAS and SG as valuation methods. For the VAS, the respondents were asked to rate health states on a scale from 0 to 100, with 0 representing the worst imaginable health and 100 indicating the best imaginable health.

For the SG, respondents were asked to make a choice between two health states (one state was a selected hypothetical scenario and the other was death). The respondents were then asked to choose between the following: they could remain in the hypothetical health state, ‘i’, for the rest of their life-time or they could receive treatment that may either restore them to full health (probability ‘p’) or immediately kill them (probability ‘1-p’). The subject continues to make choices until the preference for the two choices becomes equal. The minimum probability interval was 5%. The chances of achieving the best outcome started at 50%, and increased or decreased by 5% according to the individual’s response.

### Data analysis

First, descriptive analyses of the socio-demographic factors were performed. Next, the utility weights for each of the health states were calculated. To ensure that data from respondents who understood the valuation methods were included, 42 respondents with outlier VAS values for being dead (≥21) were excluded from the analysis, in which the methodology of exclusion was adapted from previous studies [13, 17]. The median of VAS value for being dead was 3 and its interquartile range was 8. One respondent even rated 99 for being dead.

For the VAS, the utility weight for each health state was calculated as follows: VAS value for the health state/100, where the VAS value for being dead was 0. However, if the VAS value for being dead was not 0, the following formula was used: (VAS value for the health state – the VAS value for being dead)/(100 – VAS value for being dead).

For the SG, the utility weights for each health state differed depending on the answer to the question that compared a particular health state with being dead. That is, the utility weight for each health state was calculated as the possibility of restoring full health (if the health state was regarded as a better option than being dead). The utility weight for each health state was calculated as follows: - possibility of restoring full health/(1- possibility of restoring full health), if the health state was regarded as worse than being dead. However, the utility weight for any health state that was considered worse than being dead was censored at zero.

The mean values for the utility weights according to socio-demographic factors and clinical information were compared using Student’s *t*-test and analysis of variance. Furthermore, we applied a linear regression regarding the utility weights from the VAS and SG as the dependent variable. We treated the socio-demographic factors and clinical information as independent variable. We also regarded the HPV-related health states as independent variable and created them as dummy variables with ‘HPV-positive status’ as the reference.

All statistical analyses were conducted using SPSS software (v21.0) and Stata 13.1 software (StataCorp, College Station, TX). A *P*-value < 0.05 was considered statistically significant.

## Results

### Demographic characteristics of the study subjects

Nine-hundred respondents were interviewed. The mean age of the respondents was 45.3 (standard deviation: 14.1) years, and 49.4% were male. The clinical and demographic characteristics of the respondents are shown in Table 2.

**Table 2:** Socio-demographic characteristics and clinical information for the study participants

Characteristic		Number	Percent
Gender	Male	445	49.4
	Female	455	50.6
Age group	19–29	157	17.4
	30–39	172	19.1
	40–49	197	21.9
	50–59	178	19.8
	≥60	196	21.8
Education level	Elementary school or below	29	3.2
	Middle school	80	8.9
	High school	586	65.1
	College or above	205	22.8
Occupation	Non-manual	172	19.1
	Manual	437	48.6
	Other	291	32.3
Monthly household income	Less than 2.5 million won	201	22.3
	2.5–5.0 million won	591	65.7
	>5.0 million won	108	12.0
Ambulatory care visit in the past 2 weeks	Yes	124	13.8
	No	776	86.2
Hospitalised in the past 12 months	Yes	24	2.7
	No	876	97.3
Morbidity^a^	Yes	122	13.6
	No	778	86.4

^a^Defined as the presence of any disease in the participants

### VAS and SG data

*VAS:* the highest utility weight (0.73) was assigned to HPV-positive status, followed by condyloma (0.66), and CIN grade I (0.61). The lowest utility weight (0.18) was assigned to cervical cancer requiring chemotherapy without surgery, followed by cervical cancer requiring chemoradiation therapy (0.42), and cervical cancer requiring radical hysterectomy and subsequent radiotherapy (and chemotherapy) (0.50). The difference between the utility weights for the two CIN states was only 0.01 (Table 3).

**Table 3:** Utility weights derived from the VAS and SG

Scenario		1. HPV-positive	2. CIN grade I	3. CIN grade II/III	4. Cervical cancer (simple or radical hysterectomy)	5. Cervical cancer (radical hysterectomy + radiotherapy + (chemotherapy))	6. Cervical cancer (chemoradiation)	7. Cervical cancer (chemotherapy)	8. Condyloma
VAS	N	415	475	431	439	415	412	418	427
	Mean	0.73	0.61	0.60	0.59	0.50	0.42	0.18	0.66
	SD	0.15	0.17	0.18	0.19	0.18	0.15	0.15	0.18
	Median	0.78	0.64	0.63	0.61	0.53	0.43	0.14	0.70
	1st quartile	0.67	0.50	0.49	0.47	0.37	0.33	0.07	0.55
	3rd quartile	0.84	0.72	0.73	0.72	0.63	0.50	0.25	0.80
SG	N	535	538	535	542	542	538	544	516
	Mean	0.83	0.774	0.773	0.71	0.65	0.60	0.43	0.78
	SD	0.26	0.27	0.28	0.32	0.31	0.28	0.32	0.29
	Median	0.95	0.90	0.90	0.85	0.78	0.70	0.40	0.90
	1st quartile	0.80	0.70	0.70	0.45	0.40	0.35	0.10	0.70
	3rd quartile	1.00	0.95	0.95	0.95	0.90	0.80	0.70	1.00

*HPV* human papilloma virus, *CIN* cervical intraepithelial neoplasia, *VAS* visual analogue scale, *SD* standard deviation, *SG* standard gamble

*SG:* The highest utility weight (0.83) was assigned to HPV-positive status, followed by condyloma (0.78), and CIN I (0.774). The lowest utility weight (0.43) was assigned to cervical cancer requiring chemotherapy without surgery, followed by cervical cancer requiring chemoradiation therapy (0.60), and cervical cancer requiring radical hysterectomy and subsequent radiotherapy (and chemotherapy) (0.65). The difference between the utility weights for the two CIN states was only 0.001 (Table 3).

For each health state, the mean utility weight calculated from the SG was greater than that calculated from the VAS. The difference in the utility weight calculated from the two methods was the smallest for HPV-positive status (0.10) and greatest for cervical cancer requiring chemotherapy without surgery (0.25). The mean utility weights calculated from the VAS and SG did not differ significantly according to gender, age, or educational level (Table 4). However, the respondents with a low income tended to score lower than those with a high income in both the VAS and SG, in general. For the clinical information, the particular trends could not be noticed in the utility weights from VAS and SG.

**Table 4:** Utility weights derived from the VAS and SG according to socio-demographic factors and clinical information

		Scenario 1		Scenario 2		Scenario 3		Scenario 4		Scenario 5		Scenario 6		Scenario 7		Scenario 8	
		VAS	SG	VAS	SG	VAS	SG	VAS	SG	VAS	SG	VAS	SG	VAS	SG	VAS	SG
Gender	Male	0.74	0.84	0.60	0.78	0.59	0.77	0.59	0.70	0.50	0.64	0.41	0.59	0.18	0.41	0.67	0.78
	Female	0.73	0.82	0.61	0.77	0.61	0.77	0.59	0.71	0.51	0.67	0.43	0.60	0.18	0.44	0.66	0.77
Age group	19–29	0.73	0.85	0.61	0.78	0.60	0.81	0.60	0.76	0.46	0.70	0.43	0.65	0.17	0.44	0.63	0.82
	30–39	0.76	0.84	0.59	0.76	0.60	0.80	0.59	0.71	0.52	0.64	0.43	0.57	0.20	0.43	0.67	0.77
	40–49	0.72	0.85	0.62	0.78	0.62	0.75	0.60	0.67	0.50	0.63	0.41	0.58	0.18	0.42	0.66	0.76
	50–59	0.75	0.84	0.61	0.80	0.58	0.78	0.57	0.73	0.51	0.69	0.42	0.61	0.19	0.45	0.66	0.82
	≥60	0.71	0.79	0.61	0.75	0.62	0.73	0.60	0.67	0.52	0.62	0.42	0.59	0.17	0.39	0.69	0.72
Education level	High school or below	0.73	0.83	0.61	0.77	0.61	0.77	0.59	0.69	0.51	0.66	0.43	0.60	0.18	0.42	0.67	0.77
	College or above	0.76	0.84	0.61	0.80	0.60	0.79	0.59	0.74	0.48	0.61	0.40	0.59	0.17	0.43	0.66	0.80
Occupation	Non-manual	0.77*	0.88	0.63	0.77	0.62	0.77	0.60	0.71*	0.50	0.60	0.42	0.56	0.17	0.37	0.67	0.81
	Manual	0.73*	0.82	0.59	0.77	0.61	0.76	0.58	0.67*	0.50	0.64	0.42	0.60	0.18	0.43	0.67	0.77
	Other	0.71*	0.82	0.62	0.78	0.59	0.80	0.60	0.75*	0.52	0.69	0.42	0.62	0.18	0.45	0.66	0.77
Monthly income	Less than 2.5 million won	0.71*	0.78**	0.59	0.74	0.61	0.75	0.59	0.69*	0.51	0.65*	0.40	0.59	0.16	0.41	0.69	0.72**
	2.5–5.0 million won	0.73*	0.84**	0.61	0.77	0.59	0.78	0.59	0.69*	0.50	0.64*	0.43	0.60	0.18	0.42	0.65	0.78**
	>5.0 million won	0.78*	0.90**	0.63	0.84	0.64	0.79	0.62	0.80*	0.51	0.75*	0.43	0.62	0.21	0.47	0.68	0.88**
Ambulatory care visit in past 2 weeks	Yes	0.74	0.78*	0.62	0.78	0.58	0.77	0.61	0.76	0.52	0.66	0.41	0.60	0.20	0.37	0.71*	0.71
	No	0.73	0.84*	0.61	0.77	0.61	0.77	0.59	0.70	0.50	0.65	0.42	0.60	0.18	0.43	0.66*	0.78
Hospitalised in past 12 months	Yes	0.74	0.90	0.57	0.84	0.61	0.83	0.68	0.84	0.59	0.61	0.51*	0.61	0.23	0.44	0.62	0.82
	No	0.73	0.83	0.61	0.77	0.60	0.77	0.59	0.70	0.50	0.65	0.42*	0.60	0.18	0.42	0.66	0.78
Morbidity	Yes	0.77	0.86	0.64	0.79	0.63	0.78	0.59	0.73	0.49	0.59	0.38*	0.62	0.14*	0.43	0.72*	0.77
	No	0.73	0.83	0.60	0.77	0.60	0.77	0.59	0.70	0.51	0.66	0.43*	0.59	0.19*	0.42	0.65*	0.78

*VAS* visual analogue scale, *SG* standard gamble

*P-value < 0.05; **P-value < 0.01

Table 5 shows results from the multivariable linear regression method. The health states were main relevant factors associated with the utility weights from the VAS and SG. After adjusting the respondents’ socio-demographic factors and clinical information, the utility weight for cervical cancer requiring chemotherapy without surgery were 0.554 lower than HPV-positive status in the VAS and 0.400 lower in the SG. In addition, the utility weights from respondents with a high income (>5.0 million won) were significantly higher than those with a low income (less than 2.5 million won) in both VAS and SG.

**Table 5:** Multivariable regression model for factors influencing the utility weights

Factors	Visual analogue scale			Standard gamble		
	Coefficient	95% CI coefficient		Coefficient	95% CI coefficient	
		Lower	Upper		Lower	Upper
Gender						
Men	Ref			Ref		
Women	0.006	-0.007	0.018	-0.008	-0.026	0.011
Age (years)						
19–29	Ref			Ref		
30–39	0.015	-0.004	0.034	-0.022	-0.050	0.007
40–49	0.005	-0.014	0.024	-0.038**	-0.066	-0.011
50–59	0.004	-0.016	0.023	-0.008	-0.036	0.021
≥60	0.011	-0.010	0.032	-0.075**	-0.105	-0.044
Education level						
High school or below	Ref			Ref		
College above	-0.015*	-0.031	0.000	0.003	-0.019	0.026
Occupation						
Non-manual	Ref			Ref		
Manual	-0.014	-0.031	0.003	0.019	-0.006	0.045
Other	-0.014	-0.033	0.005	0.054**	0.026	0.082
Monthly income						
Less than 2.5 million won	Ref			Ref		
2.5–5.0 million won	0.005	-0.011	0.021	-0.001	-0.024	0.022
>5.0 million won	0.034**	0.011	0.056	0.069**	0.036	0.102
Ambulatory care visit in past 2 weeks						
Yes	Ref			Ref		
No	-0.011	-0.030	0.009	0.025	-0.003	0.054
Hospitalized in past 12 month						
Yes	Ref					
No	-0.025	-0.061	0.010	-0.036	-0.091	0.018
Morbidity						
Yes	Ref			Ref		
No	0.001	-0.019	0.022	-0.030	-0.060	0.000
Scenario						
1. HPV-positive	Ref					
2. CIN grade I	-0.126**	-0.148	-0.104	-0.056**	-0.090	-0.022
3. CIN grade II/III	-0.131**	-0.153	-0.108	-0.057**	-0.091	-0.023
4. Cervical Cancer (simple or radical hysterectomy)	-0.144**	-0.166	-0.121	-0.128**	-0.162	-0.095
5. Cervical cancer (radical hysterectomy + radiotherapy + (chemotherapy))	-0.231**	-0.253	-0.208	-0.178**	-0.212	-0.145
6. Cervical cancer (chemoradiation)	-0.314**	-0.337	-0.291	-0.227**	-0.261	-0.194
7. Cervical cancer (chemotherapy)	-0.554**	-0.577	-0.531	-0.400**	-0.434	-0.367
8. Condyloma	-0.071**	-0.094	-0.048	-0.059**	-0.092	-0.025

*CI* confidence interval, *HPV* human papilloma virus, *CIN* cervical intraepithelial neoplasia

*P-value < 0.05; **P-value < 0.01

## Discussion

Here, we calculated the societal utility weights for eight hypothetical HPV-related health states based on the responses of 900 individuals selected from the general South Korean population. The main strength of this study is the fact that we evaluated the preferences of a relatively large sample (*n* = 900). A major question arises with respect to economic evaluation of healthcare programs: whose judgments should be used to define preferences? The general consensus is that the preferences of the general population should carry more weight than those of patients or healthcare professionals [21]. Other studies have examined the preferences of individuals with an abnormal Pap test [13], or the preferences of women alone [14, 15, 17]. Therefore, the results of the present study might be more appropriate for economic evaluation of cancer screening and HPV vaccination programs, for which the target population will be the general tax-paying public.

To examine the preferences of the general population, the sample group was presented with several different scenarios regarding HPV-related health states, which were designed to encompass diagnosis, treatment, and disease progression and prognosis. These scenarios were based on a literature review [13, 17] and also on patient education materials. When evaluating the preferences of the general population, these scenarios need to be explained in a manner than can be understood by all. Thus, the use of patient education material enabled people to better understand the different HPV-related health states. In particular, we included information related to prognosis, including survival rates and the chance of a complete cure. Because prognosis may play an important role in determining the utility weights of different diseases [13], the results of the present study may better reflect the preferences of the general population.

We used a VAS scale and an SG approach to determine the preferences of the study group for different HPV-related health states. The SG is a method of measuring respondents’ preferences under conditions of uncertainty, and is based directly on the von Neumann-Morgenstern utility theory, which is regarded as the gold standard for modelling rational behaviour in the context of uncertainty [22]. Furthermore, we felt that SG was more appropriate than any other evaluation method, in particular time trade-off (TTO), because assumptions based on “the rest of your life-time” are not appropriate in some scenarios; for example, “Cervical cancer requiring chemotherapy without surgery” has a 1 year survival rate of < 10%. The VAS does not allow a trade-off between different health states, and so is commonly regarded as lacking any theoretical basis when compared with choice-based methods such as SG and TTO [23]; however, the VAS is easy to understand. Thus, it is widely used in evaluation studies. Furthermore, there is no single preferred method of evaluation that is appropriate under all circumstances [24]. Therefore, it is meaningful to compare the results obtained from the VAS and SG methods.

The utility weights calculated from the VAS and SG in the present study were very consistent in terms of ordinal rankings. For example, the utility of HPV-positive status was 0.73 in the VAS and 0.83 in SG, and that for cervical cancer requiring chemotherapy without surgery was 0.18 in the VAS and 0.43 in SG. Although not directly comparable, the utility weights for HPV-related health states calculated herein (particularly those for less-serious health states) were lower than those calculated by previous studies. Kuppermann et al. reported a TTO utility of 0.909 for HPV-positive status, and 0.666 for invasive cervical cancer requiring radical hysterectomy or radiation and chemotherapy [13], whereas Melnikow et al. reported an SG utility of 0.92 for a low-grade abnormal Pap smear with cone biopsy [15]. Furthermore, Howard et al. reported an SG utility of 0.935 for HPV-positive requiring colposcopy, biopsy, and treatment [14], and Jewell EL et al. reported a TTO utility of 0.96 for minimally invasive cervical cancer requiring radical hysterectomy [17]. Marcellusi A et al. reported a TTO utility of 0.83, 0.81, and 0.58 for CIN grade I, CIN grade II/III, and cervical cancer, respectively [18]. The reason for these differences may be the detailed health state scenarios described in the present study; we provided respondents with a relatively in-depth description of the less severe HPV-related health states, including information about prognosis. This may have made respondents more fearful of such health states. In addition, there may be cultural differences in the evaluations of HPV-related health states because HPV is a sexually transmitted virus that carries considerable social stigma [25].

Another remarkable issue in this study would be gender differences in the utility weights of HPV-related health states. Utility weights for gender-specific health states, such as cervical cancer, may differ by gender, but there were no significant differences in the estimated utility weights for HPV-related health states by gender in this study. The reason for this finding is unclear, but it may be that male participants evaluated female-specific health states in this study as seriously as female participants did. In another study, utility weight lost due to anogenital warts was more prominent among women than men [18]. Furthermore, it seemed that the way of eliciting male participants’ preference regarding female-specific health states may influence gender differences. In this study, we asked male participants to imagine their mother or wife in the female-specific health states, rather than themselves in the HPV-related health states. However, further research will be needed to verify these assumptions.

The utility weights for HPV-related health states calculated in the present study are lower than those calculated in an indirect evaluation study performed in South Korea [10]. Kim et al. used a generic HRQOL instrument, the EQ-5D, to measure the HRQOL of 452 patients with CIN or cervical cancer [10]. According to that study, the highest utility weight was assigned to CIN grade I (0.937), followed by CIN grade II/III (0.933), cervical cancer (0.874), and recurrent cervical cancer (0.784). However, there are two points of concern with respect to the interpretation of those results. First, although determining the preferences of patients may reflect the actual utility, dropouts due to death or loss to follow-up and/or adaptation to disease can affect HRQOL [26]. Second, the EQ-5D, which comprises five dimensions from the HRQOL, does not fully reflect the health state of patients with CIN or cervical cancer. Thus, Kim et al. may have overestimated the utility weights [10].

There are several limitations in this study. First, we did not determine the total number of people contacted for the survey and thus we could not estimate the survey response rate. This limitation can restrict the representativeness of this study. Second, we did not ask each respondent evaluate all eight HPV-related health states. The reason for this was to minimise cognitive loading. If each respondent had to assess the utility weight for all eight HPV-related health states, the differences between the utility weights for each health state may be more marked. Third, there could have been an interviewer effect in the process of survey, which was conducted using face-to-face computer-assisted interviews. Although we trained interviewers not to have an effect on the response of survey participants, interviewers could have unintentionally affected the preferences of participants in the process of explaining HPV-related health states. Fourth, the generalizability of this study may be limited to other countries due to racial or cultural differences. Further research targeting the general public from other countries is necessary.

## Conclusion

We obtained societal utility weights for HPV-related health states from 900 respondents drawn from the general population. These results will be useful for analysing the cost-effectiveness of healthcare interventions, such as cancer screening and vaccination programs for HPV-related diseases.

## Additional files

Additional file 1: Scenarios for the different HPV-related health states. (DOCX 38 kb)
Additional file 2: Screenshot of computer-assisted questionnaire. (PPTX 8604 kb)

## Abbreviations

CIN: Cervical intraepithelial neoplasia
HPV: Human papillomavirus
HRQOL: Health-related quality of life
QALYs: Quality adjusted life years
SG: Standard gamble
TTO: Time trade-off
VAS: Visual analogue scale

## References

[1] World Health Organization, Globocan 2012: Estimated Cancer Incidence, Mortality and Prevalence Worldwide in 2012. 2012. http://globocan.iarc.fr/Pages/fact_sheets_cancer.aspx. Accessed Feb 27 2015.

[2] Elbasha EH1, Dasbach EJ, Insinga RP (2007). Model for assessing human papillomavirus vaccination strategies. Emerg Infect Dis.

[3] Villa LL, Costa RL, Petta CA, Andrade RP, Ault KA, Giuliano AR (2005). Prophylactic quadrivalent human papillomavirus (types 6, 11, 16, and 18) L1 virus-like particle vaccine in young women: a randomised double-blind placebo-controlled multicentre phase II efficacy trial. Lancet Oncol.

[4] Garland SM, Hernandez-Avila M, Wheeler CM, Perez G, Harper DM, Leodolter S (2007). Quadrivalent vaccine against human papillomavirus to prevent anogenital diseases. N Engl J Med.

[5] FUTURE II Study Group (2007). Quadrivalent vaccine against human papillomavirus to prevent high-grade cervical lesions. N Engl J Med.

[6] Ault KA, Future II Study Group (2007). Effect of prophylactic human papillomavirus L1 virus-like-particle vaccine on risk of cervical intraepithelial neoplasia grade 2, grade 3, and adenocarcinoma in situ: a combined analysis of four randomised clinical trials. Lancet.

[7] Muñoz N, Manalastas R, Pitisuttithum P, Tresukosol D, Monsonego J, Ault K (2009). Safety, immunogenicity, and efficacy of quadrivalent human papillomavirus (types 6, 11, 16, 18) recombinant vaccine in women aged 24-45 years: a randomised, double-blind trial. Lancet.

[8] Villa LL, Costa RL, Petta CA, Andrade RP, Paavonen J, Iversen OE (2006). High sustained efficacy of a prophylactic quadrivalent human papillomavirus types 6/11/16/18 L1 virus-like particle vaccine through 5 years of follow-up. Br J Cancer.

[9] Kim JJ, Goldie SJ (2009). Cost effectiveness analysis of including boys in a human papillomavirus vaccination programme in the United States. BMJ.

[10] Kim Y, Ahn J, Kim YJ, Park J, Kim J, Lee YJ, et al. Economic Evaluation of HPV Vaccination. National Evidence-based Healthcare Collaborating Agency, 2013. In Korean

[11] Sassi F (2006). Calculating QALYs, comparing QALY and DALY calculations. Health Policy Plan.

[12] Whitehead SJ, Ali S (2010). Health outcomes in economic evaluation: the QALY and utilities. Br Med Bull.

[13] Kuppermann M, Melnikow J, Slee C, Tancredi DJ, Kulasingam S, Birch S (2010). Preferences for surveillance strategies for women treated for high-grade precancerous cervical lesions. Gynecol Oncol.

[14] Howard K, Salkeld G, McCaffery K, Irwig L (2008). HPV triage testing or repeat Pap smear for the management of atypical squamous cells (ASCUS) on Pap smear: is there evidence of process utility?. Health Econ.

[15] Melnikow J, Kuppermann M, Birch S, Chan BK, Nuovo J (2002). Management of the low-grade abnormal Pap smear: What are women's preferences?. J Fam Pract.

[16] Mennini FS, Panatto D, Marcellusi A, Cristoforoni P, De Vincenzo R, Di Capua E (2011). Time trade-off procedure for measuring health utilities loss with human papillomavirus-induced diseases: a multicenter, retrospective, observational pilot study in Italy. Clin Ther.

[17] Jewell EL, Smrtka M, Broadwater G, Valea F, Davis DM, Nolte KC (2011). Utility scores and treatment preferences for clinical early-stage cervical cancer. Value Health.

[18] Marcellusi A, Capone A, Favato G, Mennini FS, Baio G, Haeussler K (2015). Health utilities lost and risk facto+rs associated with HPV-induced diseases in men and women: the HPV Italian collaborative study group. Clin Ther.

[19] Hadorn DC (1991). The role of public values in setting health care priorities. Soc Sci Med.

[20] Bae EY (2008). Guidelines for economic evaluation of pharmaceuticals in Korea. J Prev Med Public Health.

[21] Dolan P, Olsen JA, Menzel P, Richardson J (2003). An inquiry into the different perspectives that can be used when eliciting preferences in health. Health Econ.

[22] Gafni A (1994). The standard gamble method: what is being measured and how it is interpreted. Health Serv Res.

[23] Dolan P, Sutton M (1997). Mapping visual analogue scale health state valuations onto standard gamble and time trade-off values. Soc Sci Med.

[24] Salomon JA, Murray CJ (2004). A multi-method approach to measuring health-state valuations. Health Econ.

[25] Ustün TB, Rehm J, Chatterji S, Saxena S, Trotter R, Room R (1999). Multiple-informant ranking of the disabling effects of different health conditions in 14 countries. WHO/NIH Joint Project CAR Study Group. Lancet.

[26] Breetvelt IS, Van Dam FS (1991). Underreporting by cancer patients: the case of response-shift. Soc Sci Med.

