# Supplementary material for: Estimation of utility weights for human papilloma virus-related health states according to disease severity
Source: Health Qual Life Outcomes. 2016 Nov 28;14:163. doi: 10.1186/s12955-016-0566-8 (PMC5126850; doi:10.1186/s12955-016-0566-8)
Supplement: Additional file 1: — Scenarios for the different HPV-related health states. (DOCX 38 kb) [file 12955_2016_566_MOESM1_ESM.docx]

**Supplement 1. Scenarios for the different HPV-related health states**

Descriptions of the health states related to cervical cancer and human papillomavirus infection.

Below is a description of diseases associated with HPV infection. Please read the following carefully:

⦁ Pap test: This test is conducted to check for abnormal cells (e.g., cancerous cells) in the cervix. The patient lies down on a reclining chair with her legs apart. The physician inserts a vaginal speculum into the patient's vagina and softly scrapes the cervix with a cotton swab to collect a sample of cells. The procedure should be painless, but some patients may experience mild discomfort, and some patients may feel some degree of embarrassment as well. There may be some secretion after the examination.

⦁ Human papilloma virus test: This test is conducted to check for the presence of viruses (commonly transmitted by sexual contact) that may cause cervical cancer. The patient lies down on a reclining chair with her legs apart. The physician inserts a vaginal speculum into the patient's vagina and softly scrapes the cervix with a cotton swab to collect a sample of cells. The procedure should be painless, but some patients may experience mild discomfort, and some patients may feel some degree of embarrassment as well. There may be some secretion after the examination.

⦁ Colposcopy: This test is generally conducted to examine the cervix more thoroughly if an abnormality is found in the Pap test. The patient lies down on a reclining chair with her legs apart. The physician inserts a vaginal speculum into the patient's vagina and some drugs are applied to the cervix to help examine for areas of abnormality. The procedure should be painless, but some patients may experience mild discomfort, and some patients may feel some degree of embarrassment as well. There may be some secretion after the examination.

⦁ Biopsy: If abnormal tissue is detected during a colposcopy, the doctor may perform a biopsy. Local anaesthesia is usually used, but patients often experience pain. There may also be some pain and bleeding after the biopsy. Sexual intercourse should be avoided for about 1 week after the biopsy.

**Scenarios**

**1) Normal cervix, HPV-positive**

- Diagnosis: You have recently undergone a Pap test and a HPV test. After 1–2 weeks, your Pap test came back as normal, but the HPV test came back as positive.

- Symptoms: There are no specific symptoms.

- Treatment: The HPV vaccine may be used to treat cases of asymptomatic HPV infection, but generally, the infection is monitored without treatment. You should have follow-up Pap and HPV tests in 3 months.

- Progress and prognosis of the disease: You are worried that the HPV infection may cause cancer, but in 90% of cases everything returns to normal within 2 years without treatment.

**2) Cervical intraepithelial neoplasia (CIN) grade I**

- Diagnosis: Abnormal tissue was detected during a recent Pap test and colposcopy; therefore, a biopsy was performed. You were then diagnosed with CIN I.

- Symptoms: There are no specific symptoms.

- Treatment: You may not receive treatment or you may receive cervical conisation. Cervical conisation refers to the excision of a cone-shaped sample of tissue from the cervix under intravenous anaesthesia. You should have follow-up Pap and HPV tests in 3 months.

- Progress and prognosis of the disease: You are worried that the HPV infection may cause cancer; however, in more than 90% of cases it does not progress and things returns to normal on their own. However, recurrence may happen in some cases.

**3) Cervical intraepithelial neoplasia (CIN) grade II/III**

- Diagnosis: Abnormal tissue was identified during a recent Pap test and colposcopy; therefore, a biopsy was performed. You were then diagnosed with CIN II/III.

- Symptoms: There are no specific symptoms.

- Treatment: You may undergo cervical conisation. Cervical conisation refers to the excision of a cone-shaped sample of tissue from the cervix under intravenous anaesthesia. You should have follow-up Pap and HPV tests in 3 months. Although there are few complications, bleeding or increased vaginal secretion may occur. Rarely, infection or cervical incompetence may occur.

- Progress and prognosis of the disease: You are worried that the HPV infection may cause cancer; however, in more than 90% of cases it does not progress and things go back to normal on their own. However, recurrence may happen in some cases.

**4) Cervical cancer requiring simple or radical hysterectomy**

- Diagnosis: Abnormal tissue was found during a recent Pap test and colposcopy; therefore, a biopsy was performed. You were diagnosed with cervical cancer requiring a hysterectomy.

- Symptoms: There are no specific symptoms, but there may be minor vaginal bleeding after intercourse. In some cases, bleeding and vaginal secretion may increase. In addition, ulcers may become worse or the infection may cause an unpleasant smell.

- Treatment: You require a simple or radical hysterectomy. A simple hysterectomy involves removing the uterus and cervix under general anaesthesia. A radical hysterectomy involves removal of the ovaries, lymph nodes, and part of the vagina, in addition to a simple hysterectomy. A hysterectomy can be performed in different ways (laparoscopic surgery or open abdominal surgery) and you can expect to be in hospital for about 10 days. Complications include infection, damage to other organs, or bleeding. Complete resection should be confirmed by pathology examination performed at 1–2 weeks after surgery. During the first 3–6 months after surgery, you should have regular follow-up visits. The intervals between visits will become longer over time.

- Progress and prognosis of the disease: After laparoscopic surgery you will have scars (about 2.5 cm) at three or four incision sites. After abdominal surgery, you will have a scar (about 15 cm) across the bottom of the abdomen. The surgery will bring on a menopause and, in some cases, you may not be able to have children. You are likely to experience a number of emotions, from fear of recurrent cancer or death from cancer to depression. In some cases, patients experience problems with their sex life, swelling of the legs, problems with urination and defecation, or nerve stimulation. The chance of a complete cure is 90%.

**5) Cervical cancer requiring a radical hysterectomy and consequent radiotherapy (and chemotherapy)**

- Diagnosis: Abnormal tissue was detected during a recent Pap test and colposcopy; therefore, a biopsy was performed. You were diagnosed with cervical cancer requiring a radical hysterectomy and subsequent radiotherapy (and chemotherapy).

- Symptoms: There are no specific symptoms, but you may experience mild slight vaginal bleeding after intercourse. In some cases, bleeding and vaginal secretions may increase. In addition, ulcers can become worse, or the infection may cause an unpleasant smell.

- Treatment: First, you need a radical hysterectomy. A radical hysterectomy involves removal of the uterus, cervix, ovaries, lymph nodes and part of the vagina under general anaesthesia. A hysterectomy can be performed in different ways (laparoscopic surgery or open abdominal surgery) and you can expect to be in hospital for about 10 days. Complications include infection, damage to other organs, or bleeding. Because a high risk of recurrence was confirmed by pathology examination performed at 1–2 weeks after surgery, you will receive radiation (and chemotherapy) treatment 4 weeks after surgery, lasting for 2 months. Options for radiation therapy include brachytherapy, where a radiation source is placed inside the vagina, and external beam radiation therapy. In the first 3–6 months after surgery, you should have regular follow-up visits. The intervals between these visits will gradually increase.

- Progress and prognosis of the disease: After laparoscopic surgery you will have scars (about 2.5 cm) at three or four incision sites. After abdominal surgery, you will have a scar (about 15 cm) across the bottom of the abdomen. The surgery will bring on a menopause and, in some cases, you may not be able to have children. You are likely to experience a number of emotions, from fear of recurrent cancer or death from cancer to depression. In some cases, patients experience problems with their sex life, swelling of the legs, problems with urination and defecation, or nerve stimulation. Atrophy of the vaginal mucosa can occur due to radiation, and there may be a reduction in the secretion of sex hormones. Nausea and vomiting are common side effects of chemotherapy; hair loss can also occur. The chance of a complete cure is 80%.

**6) Cervical cancer requiring chemoradiation therapy**

- Diagnosis: Abnormal tissue was detected during a recent Pap test and colposcopy; therefore, a biopsy was performed. You were diagnosed with cervical cancer that requires chemoradiation therapy.

- Symptoms: There are no specific symptoms, but you may experience slight vaginal bleeding after intercourse. In some cases, bleeding and vaginal secretion may increase as the cancer progresses. In addition, ulcers can worsen or the infection can cause an unpleasant smell. If the cancer is more advanced, it may invade surrounding organs, including the rectum, bladder, urethra, pelvis, and nerves. In this case, you may experience problems with urination, defecation, blood in the urine, rectal bleeding, back pain, pain in the lower extremities, swelling, weight loss, and other symptoms.

- Treatment: Because the cancer is advanced, you receive chemoradiation therapy without surgery. The mean duration of treatment is about 10 weeks (2.5 months). Options for radiation therapy include brachytherapy, where a radiation source is placed inside the vagina, and external beam radiation therapy. Chemotherapy involves the intravenous injection of anti-cancer drugs. You should attend regular follow-up visits during the first 3–6 months after surgery; the intervals between visits will increase over time.

- Progress and prognosis of the disease: You are likely to experience a number of emotions, from fear of recurrent cancer or death from cancer to depression. Atrophy of the vaginal mucosa can occur due to radiation, and there may be a reduction in the secretion of sex hormones. Nausea and vomiting are common side effects of chemotherapy; hair loss can also occur. The chance of a complete cure is 60%.

**7) Cervical cancer requiring chemotherapy without surgery**

- Diagnosis: Abnormal tissue was detected during a recent Pap test and colposcopy; therefore, a biopsy was performed. As a result, you were diagnosed with cervical cancer requiring chemotherapy without surgery.

- Symptoms: You may have experienced a slight vaginal bleeding after intercourse. In some cases, bleeding and vaginal secretion may increase as the cancer progresses. In addition, ulcers can worsen, or the infection may cause an unpleasant smell. If the cancer is more advanced, it may invade the surrounding organs, including the rectum, bladder, urethra, pelvis and nerves. In this case, you may experience problems with urination and defecation, blood in the urine, rectal bleeding, back pain, pain in the lower extremities, swelling, weight loss, and other symptoms.

- Treatment: Because the cancer is advanced, you receive chemotherapy without surgical treatment. Because the survival rate is usually less than 10%, the treatment period is usually less than 1 year. Chemotherapy involved the intravenous injection of anti-cancer drugs.

- Progress and prognosis of the disease: You are likely to experience a number of emotions, from fear of recurrent cancer or death from cancer to depression. Nausea and vomiting are common side effects of chemotherapy; hair loss may also occur. Depending on the degree of cancer progression, daily activities may be difficult. The 1 year survival rate is less than 10%.

**8) Condyloma (genital warts [condyloma acuminatum])**

- Diagnosis: A recent examination revealed white or pink grainy papules (small solid elevated areas of skin) on the external genitalia, and you have undergone blood tests (e.g., for syphilis) and a physical examination. You were diagnosed with condyloma.

- Symptoms: Condyloma is usually caused by HPV infection. There may be no specific symptoms, but you may experience vaginal bleeding or secretions.

- Treatment: Depending on the shape, location, size, and number of condyloma, you may receive medical or surgical treatment. Medical treatment involves the topical application of drugs directly to the affected area. Usually, treatment lasts for 3 months and is carried out on an outpatient basis. Surgical treatment involves cryotherapy or carbon dioxide laser therapy, both of which last for 1 week.

- Progress and prognosis of the disease: The lesions are not noticeable externally, but due to their appearance they may cause aversion. In addition, there is some concern about genital warts because they may progress to genital cancers. The side effects associated with medical treatment may include skin irritation, depending on the drug. Surgical treatment may be painful and, in rare cases, infection may occur. Condyloma may disappear without treatment; however, treatment is usually required. In most cases, it disappears within 3 months, although recurrence is noted in 30% of cases.
