# Supplementary material for: Estimation of utility weights for human papilloma virus-related health states according to disease severity
Source: Health Qual Life Outcomes. 2016 Nov 28;14:163. doi: 10.1186/s12955-016-0566-8 (PMC5126850; doi:10.1186/s12955-016-0566-8)
Supplement: Additional file 2: — Screenshot of computer-assisted questionnaire. (PPTX 8604 kb) [file 12955_2016_566_MOESM2_ESM.pptx]

## Slide 1
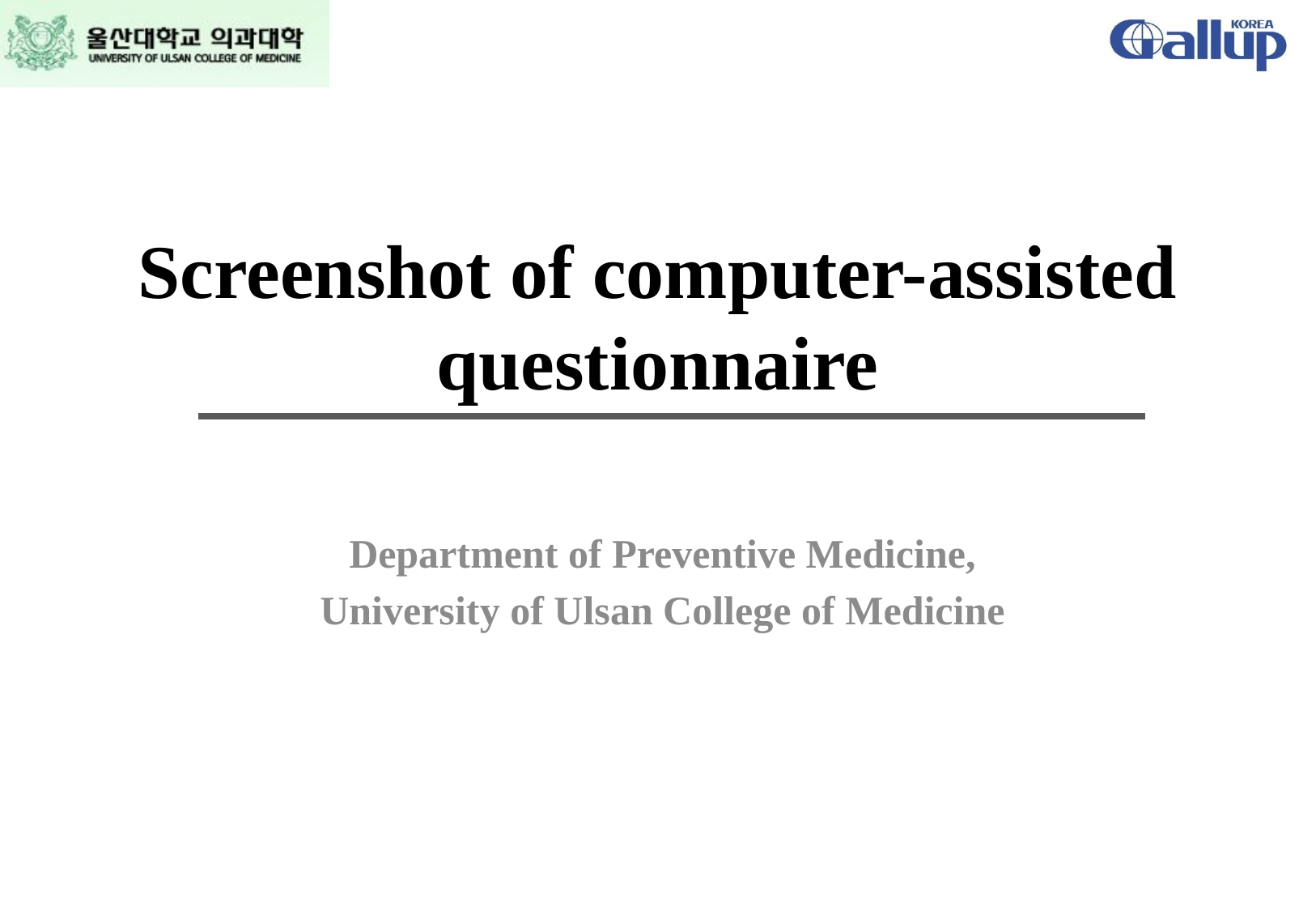

Screenshot of computer-assisted questionnaire
Department of Preventive Medicine,
University of Ulsan College of Medicine

## Slide 2
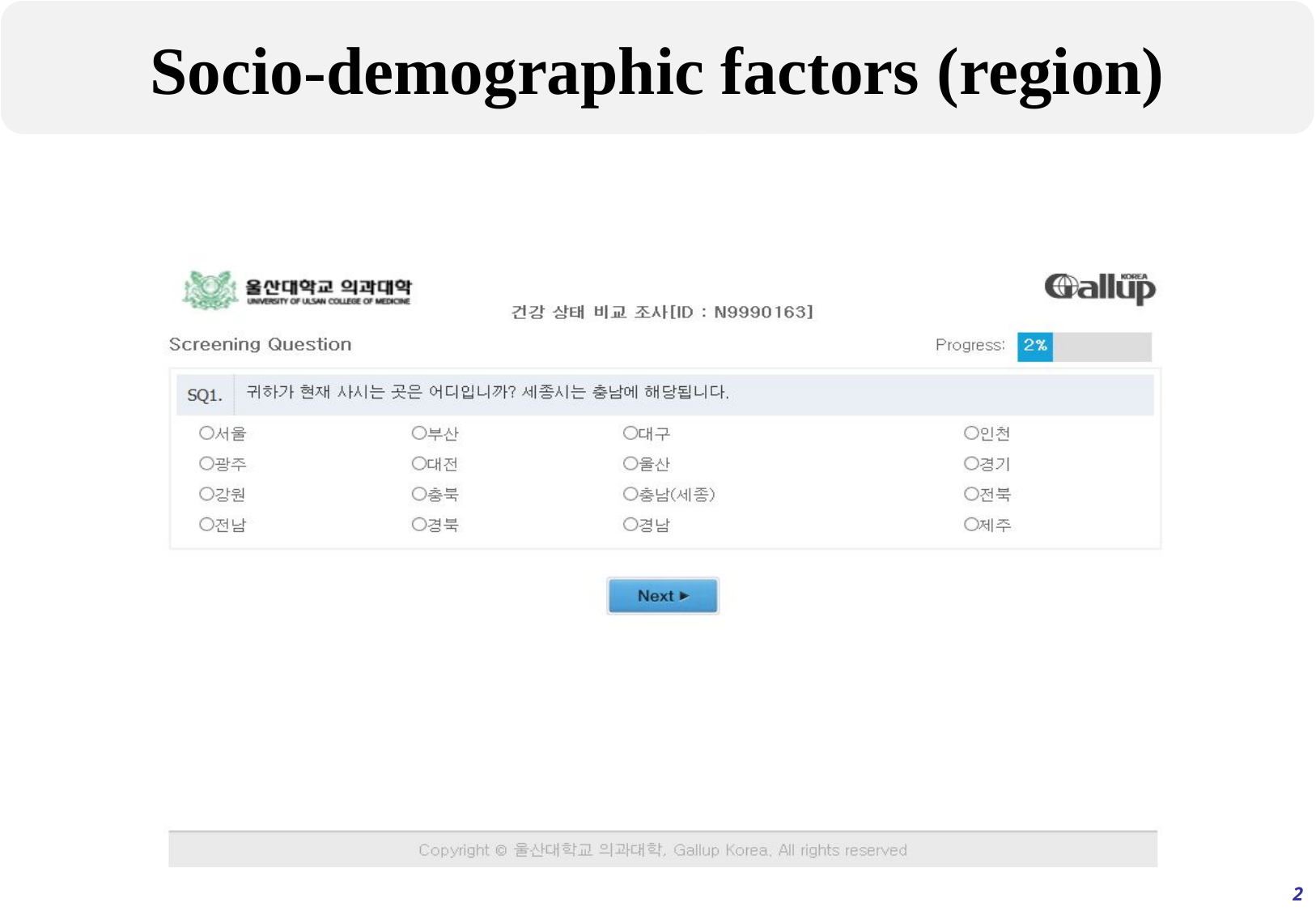

Socio-demographic factors (region)

## Slide 3
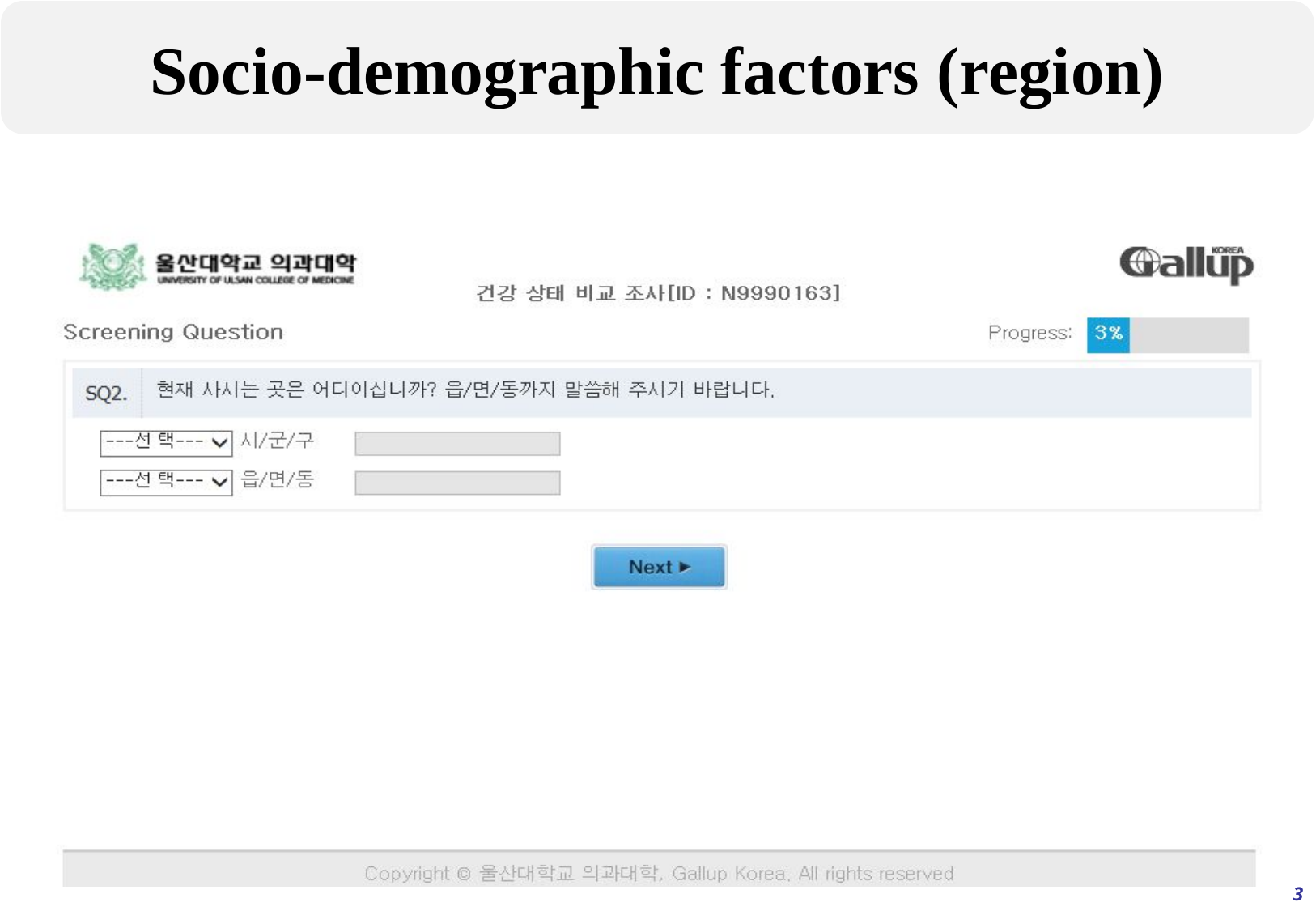

Socio-demographic factors (region)

## Slide 4
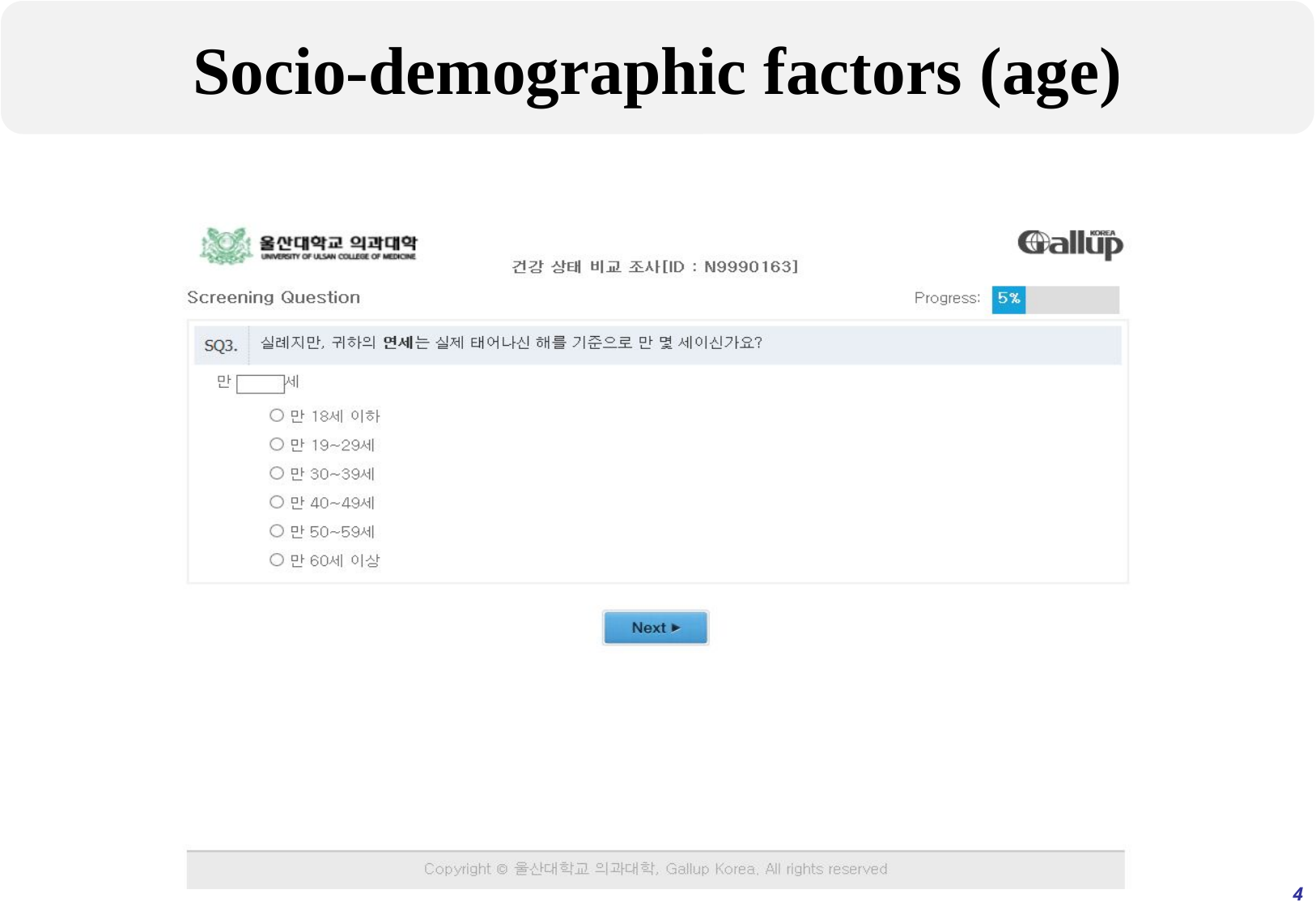

Socio-demographic factors (age)

## Slide 5
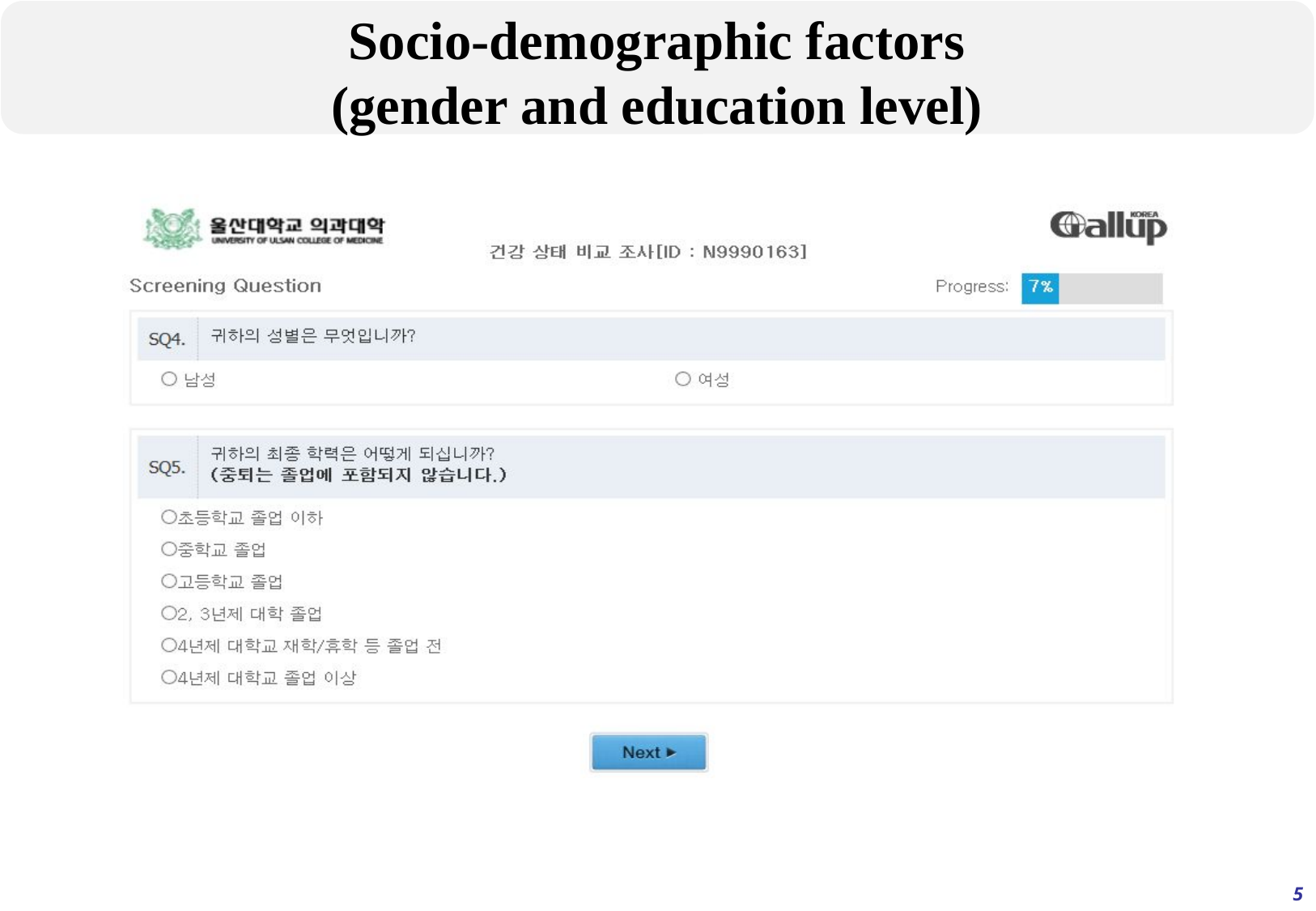

Socio-demographic factors
(gender and education level)

## Slide 6
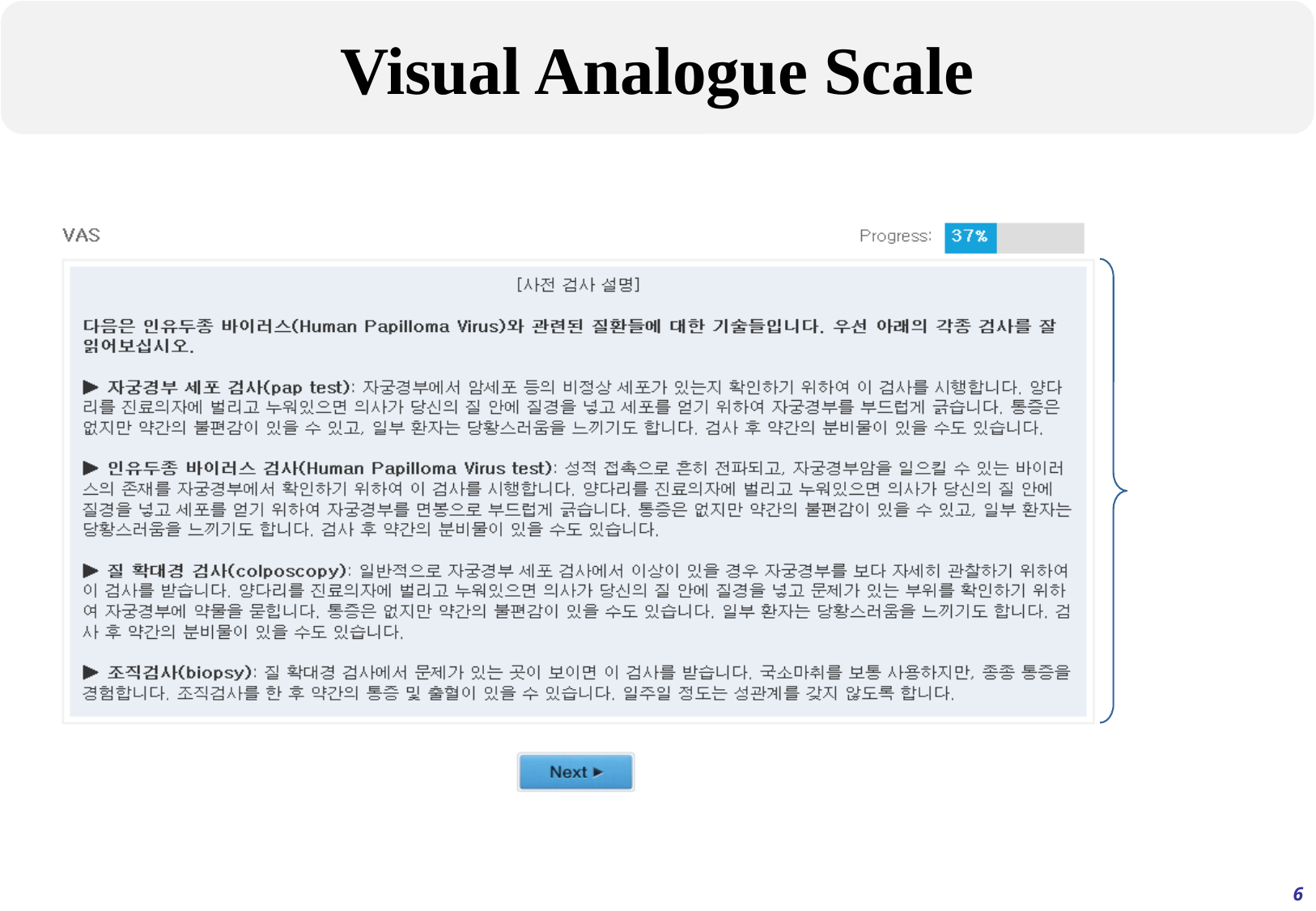

Visual Analogue Scale

## Slide 7
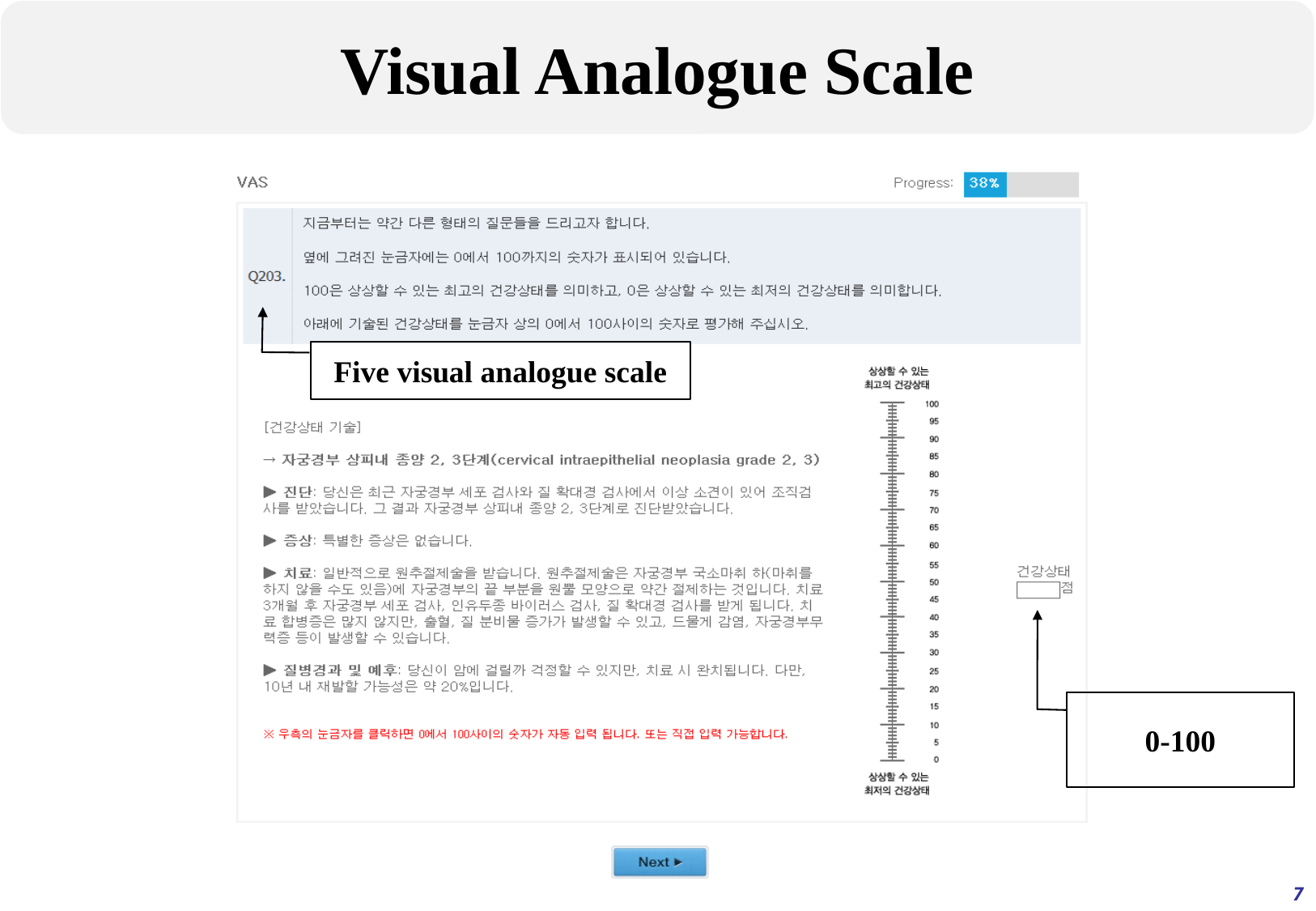

Visual Analogue Scale
Five visual analogue scale
0-100

## Slide 8
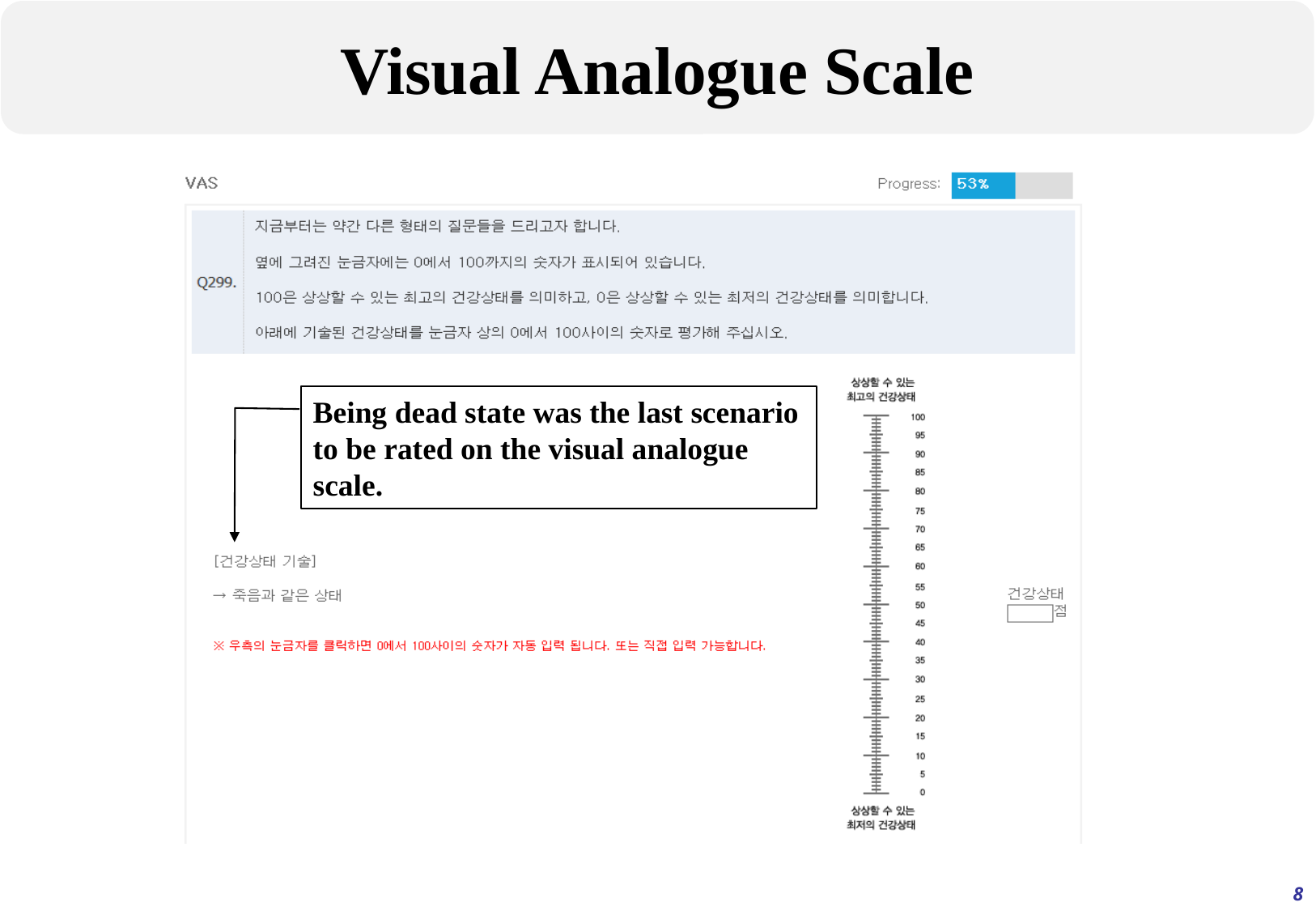

Visual Analogue Scale
Being dead state was the last scenario to be rated on the visual analogue scale.

## Slide 9
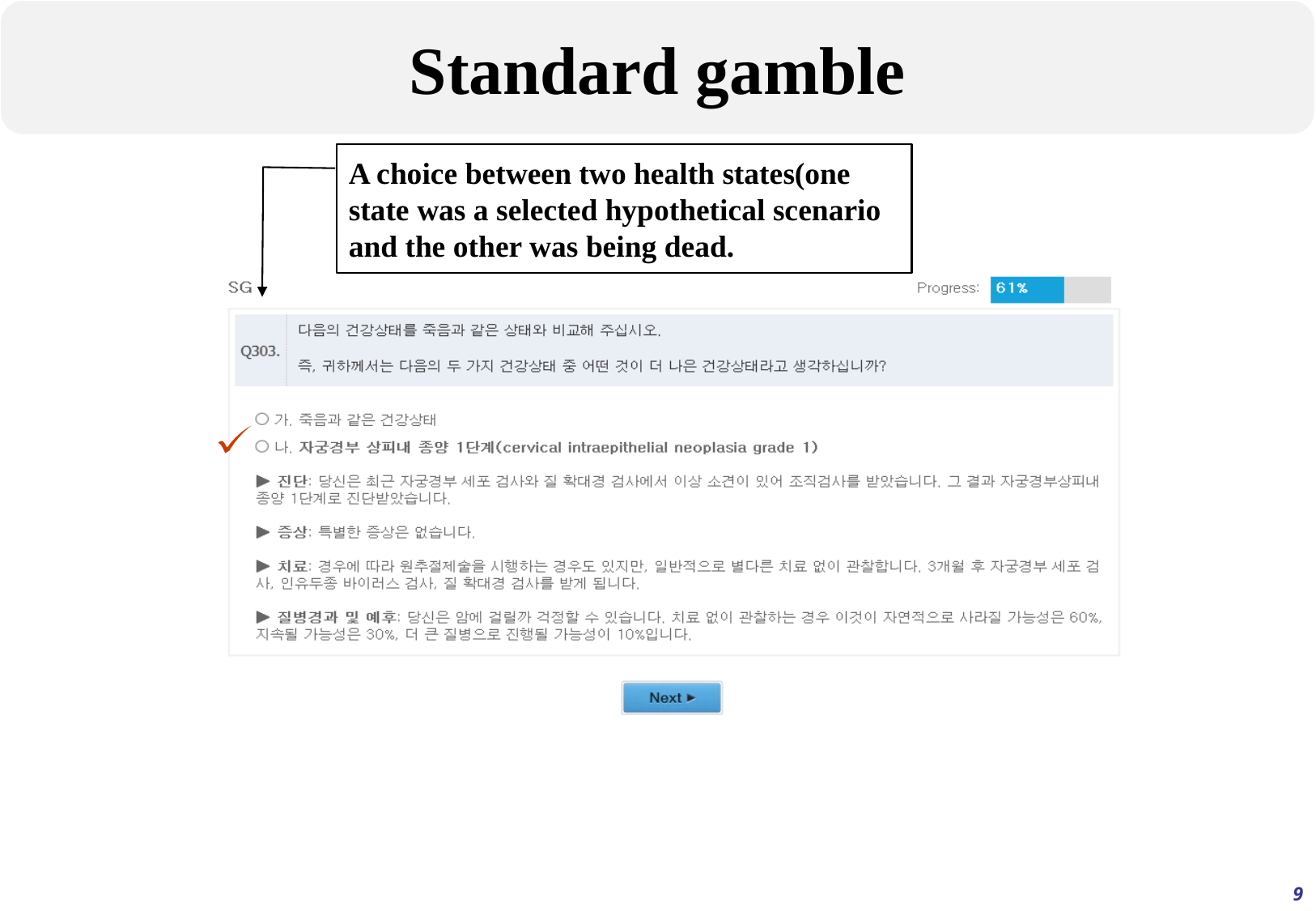

Standard gamble
A choice between two health states(one state was a selected hypothetical scenario and the other was being dead.

## Slide 10
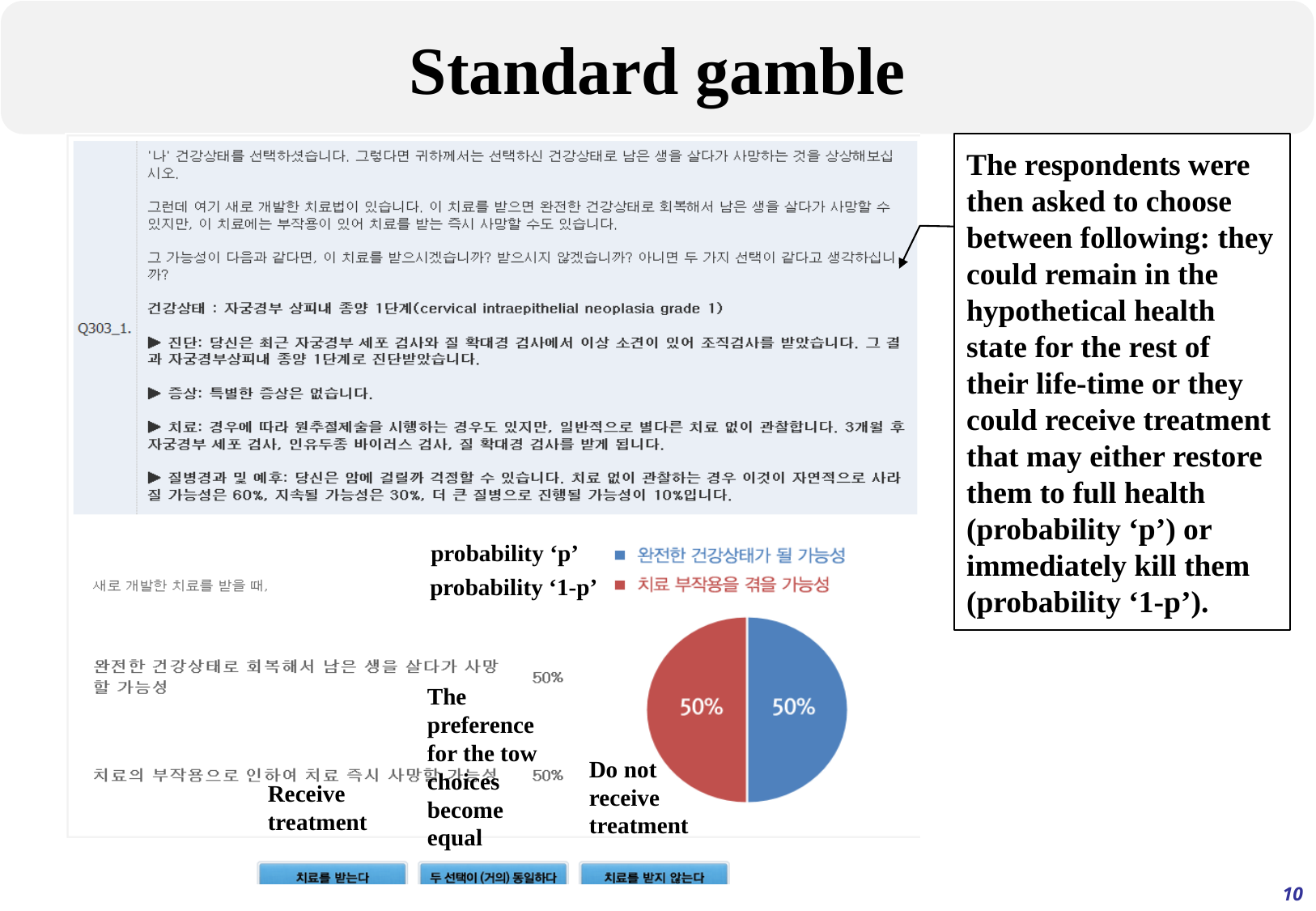

Standard gamble
The respondents were then asked to choose between following: they could remain in the hypothetical health state for the rest of their life-time or they could receive treatment that may either restore them to full health (probability ‘p’) or immediately kill them (probability ‘1-p’).
probability ‘p’
probability ‘1-p’
The preference for the tow choices become equal
Do not receive treatment
Receive treatment

## Slide 11
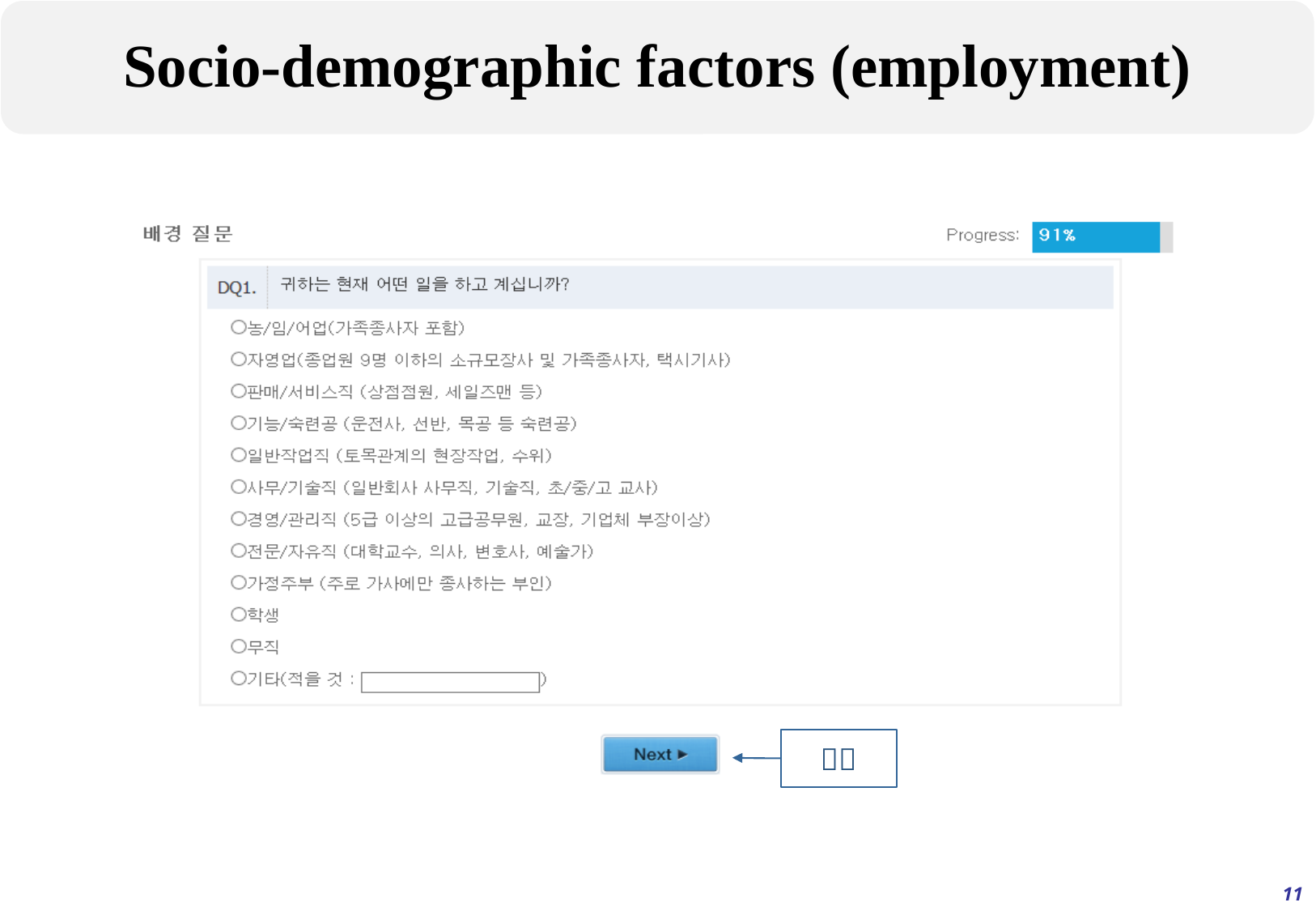

Socio-demographic factors (employment)
클릭

## Slide 12
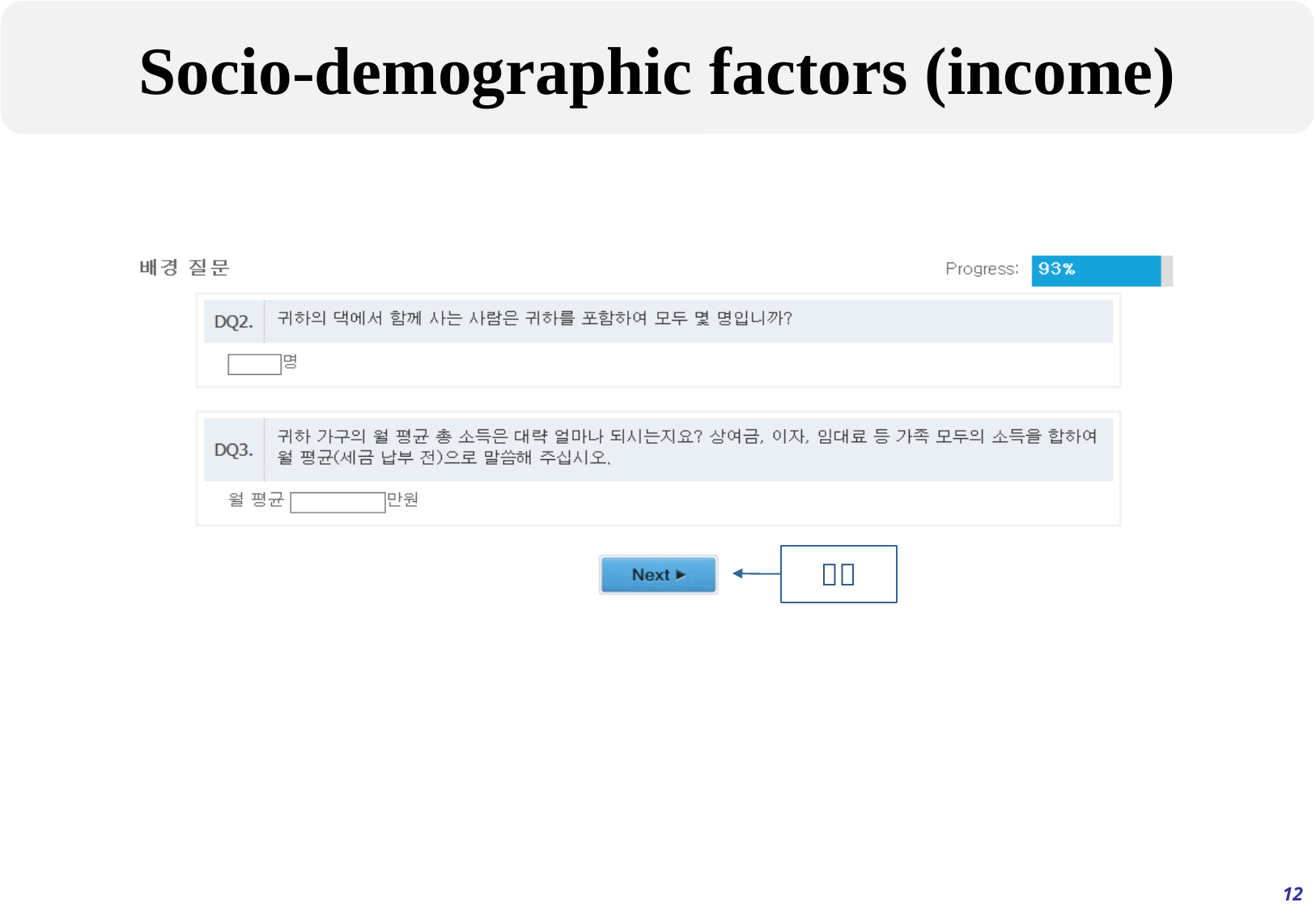

Socio-demographic factors (income)
클릭

## Slide 13
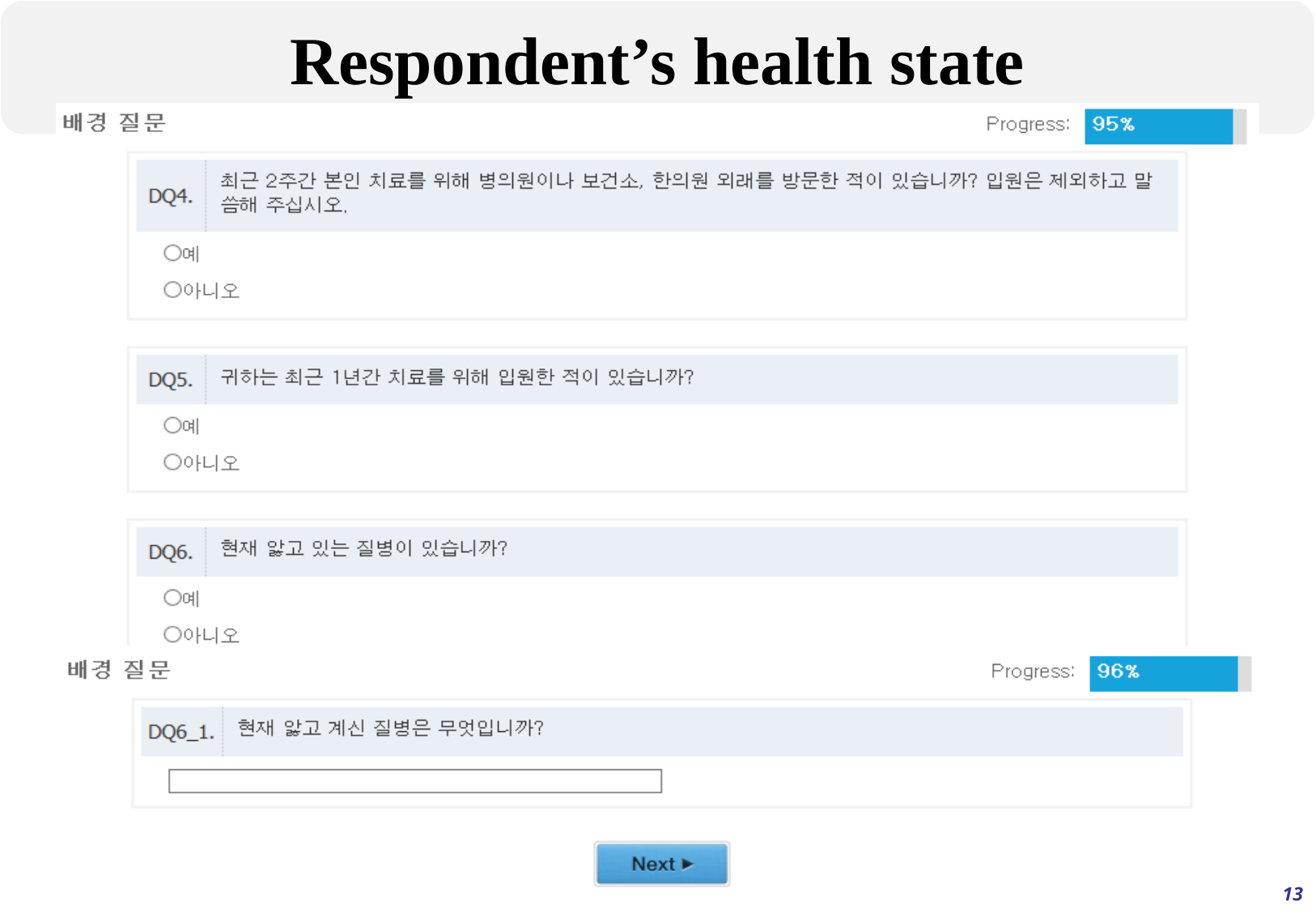

Respondent’s health state

## Slide 14
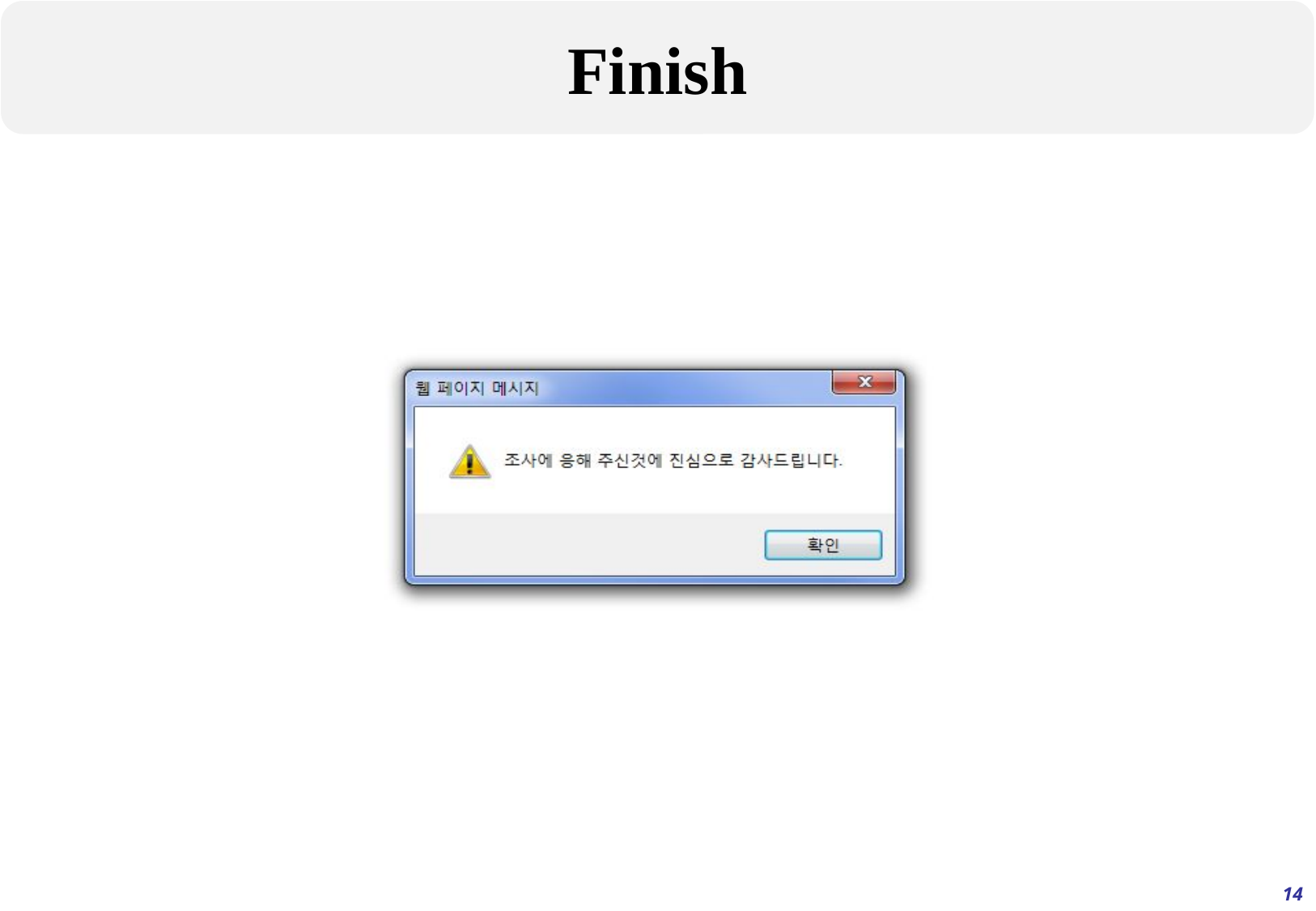

Finish

## Slide 15
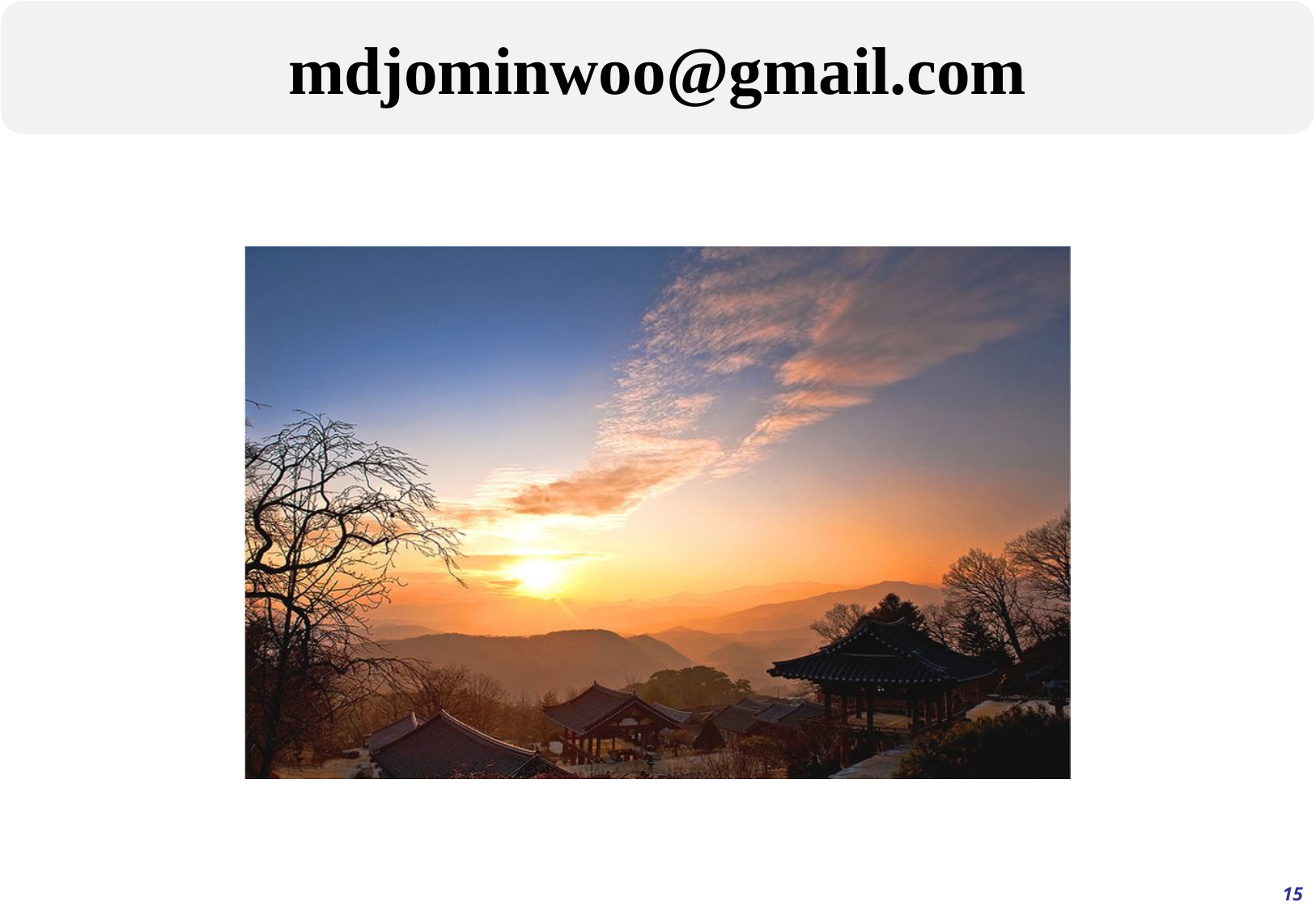

mdjominwoo@gmail.com
